# β-Arrestin Interacts with the Beta/Gamma Subunits of Trimeric G-Proteins and Dishevelled in the Wnt/Ca^2+^ Pathway in Xenopus Gastrulation

**DOI:** 10.1371/journal.pone.0087132

**Published:** 2014-01-29

**Authors:** Katharina Seitz, Verena Dürsch, Jakub Harnoš, Vitezslav Bryja, Marc Gentzel, Alexandra Schambony

**Affiliations:** 1 Biology Department, Developmental Biology, Friedrich-Alexander University Erlangen-Nuremberg, Erlangen, Germany; 2 Institute of Experimental Biology, Faculty of Science, Masaryk University, Brno, Czech Republic; 3 Institute of Biophysics, Academy of Sciences of the Czech Republic, Brno, Czech Republic; 4 Max Planck Institute of Molecular Cell Biology and Genetics, Dresden, Germany; The Rockefeller University, United States of America

## Abstract

β-Catenin independent, non-canonical Wnt signaling pathways play a major role in the regulation of morphogenetic movements in vertebrates. The term non-canonical Wnt signaling comprises multiple, intracellularly divergent, Wnt-activated and β-Catenin independent signaling cascades including the Wnt/Planar Cell Polarity and the Wnt/Ca^2+^ cascades. Wnt/Planar Cell Polarity and Wnt/Ca^2+^ pathways share common effector proteins, including the Wnt ligand, Frizzled receptors and Dishevelled, with each other and with additional branches of Wnt signaling. Along with the aforementioned proteins, β-Arrestin has been identified as an essential effector protein in the Wnt/β-Catenin and the Wnt/Planar Cell Polarity pathway. Our results demonstrate that β-Arrestin is required in the Wnt/Ca^2+^ signaling cascade upstream of Protein Kinase C (PKC) and Ca^2+^/Calmodulin-dependent Protein Kinase II (CamKII). We have further characterized the role of β-Arrestin in this branch of non-canonical Wnt signaling by knock-down and rescue experiments in Xenopus embryo explants and analyzed protein-protein interactions in 293T cells. Functional interaction of β-Arrestin, the β subunit of trimeric G-proteins and Dishevelled is required to induce PKC activation and membrane translocation. In Xenopus gastrulation, β-Arrestin function in Wnt/Ca^2+^ signaling is essential for convergent extension movements. We further show that β-Arrestin physically interacts with the β subunit of trimeric G-proteins and Dishevelled, and that the interaction between β-Arrestin and Dishevelled is promoted by the beta/gamma subunits of trimeric G-proteins, indicating the formation of a multiprotein signaling complex.

## Introduction

Wnt signaling plays a crucial role in pattern formation, tissue specification and cellular organization during embryogenesis. Wnt signaling pathways are generally subdivided into the canonical Wnt pathway, which leads to stabilization and nuclear translocation of β-Catenin, and the more divergent non-canonical, β-Catenin independent pathways. Both β-Catenin dependent and independent Wnt pathways are activated by heteromeric receptor complexes of Frizzled family seven-pass transmembrane receptors and LRP5/6 or PTK7/Ryk/Ror2 co-receptors, respectively. The β-Catenin independent Wnt pathways encompass a number of biochemically and functionally distinct signaling cascades, including the Wnt/PCP and the Wnt/Ca^2+^ pathways. Wnt/PCP signaling leads to a Dishevelled (Dvl) mediated activation of Rho family small GTPases (RhoA, Rac1) and subsequent activation and phosphorylation of c-Jun N-terminal Kinase (JNK [1]). The Wnt/Ca^2+^ pathway is characterized by Gα_i/o_-triggered, Pertussis Toxin (PTX)-sensitive calcium release and activation of Ca^2+^-regulated effector proteins, including Protein Kinase C alpha (PKCα), Ca^2+^/Calmodulin-dependent Protein Kinase II (CamKII) and Nuclear Factor of Activated T-Cells (NFAT [2]).

In multiple developmental processes, including gastrulation and cardiac development, Wnt/PCP and Wnt/Ca^2+^ signaling are required simultaneously and are often activated by the same Wnt ligand and Frizzled receptor. Wnt-11 and Frizzled 7 activated PKC signaling is required for tissue separation of mesoderm and ectoderm during gastrulation [3]. In the mesoderm, Wnt-11, Frizzled 7 (Fzd7), Dvl2 and β-Arrestin2 (Arrb2) activate Wnt/PCP signaling during convergent extension movements [4]–[7]. Similarly, Wnt-11 mediates both Wnt/Ca^2+^ and Wnt/PCP signaling in cardiac development [8], [9]. Recent reviews have depicted Wnt signaling cascades as a signaling network rather than as distinct signaling cascades [10], however, the biochemical interaction and integration of these different branches of non-canonical Wnt signaling is yet unclear.

β-Arrestins were initially described as proteins involved in desensitizing and endocytosis of G-protein coupled receptors (GPCRs [11]). More recent studies revealed that β-Arrestins play a role in multiple signal transduction pathways, including canonical and non-canonical Wnt signaling (for review [11], [12]). In contrast to classical GPCR desensitizing, β-Arrestin2 acts as a positive regulator in Wnt signaling. We have previously shown that β-Arrestin2 interacts with Dvl and is required for Wnt/β-Catenin signal transduction [13]. Others and we have also described a role for β-Arrestin2 in the activation of the PCP pathway [7], [14]. Here we show that β-Arrestin2 is required in the Wnt/Ca^2+^ signaling cascade; it interacts functionally and physically with the β subunit of trimeric G-proteins and Dishevelled, which are both known effectors in the Wnt/Ca^2+^ cascade [15], [16]. We show further that Wnt/Ca^2+^ signaling is essential for proper convergent extension movements in Xenopus embryos and that β-Arrestin2 functionally links Wnt/Ca^2+^ and Wnt/PCP signaling in convergent extension movements.

## Materials and Methods

### Xenopus laevis Embryos

Xenopus embryos were generated and cultured according to general protocols and staged according to the normal table of Nieuwkoop and Faber [17]. All procedures were performed according to the German animal use and care law (Tierschutzgesetz) and approved by the German state administration Bavaria (Regierung von Mittelfranken).

### Cell Culture and Transfection

293T human embryonic kidney cells (Leibniz Institute Collections of Microorganisms and Cell Culture, DSMZ, Germany) were cultured in DMEM supplemented with 10% fetal calf serum (Life Technologies, CA, USA) at 37°C in a humidified atmosphere of 10% CO_2_. Plasmid transfections were performed using either TransPassD2 (New England Biolabs, MA, USA) or Nanofectin (GE Healthcare, Freiburg, Germany) according to the manufacturers' protocols.

### Plasmids and Morpholinos

The following plasmids and Morpholinos have been described previously: pcDNA-*flag*-*arrb2* [13], pCS2+ *fzd7*, pCS2+ *rhoA V12*, pCS2+ *rac1 V14* [18], pCS2+ *cdc42* [19]; Arrb2 Morpholino 1 (Arrb2 MO1, [13]), Fzd7 Morpholino [3], Dvl2 MO [7], Dvl1 MO, Dvl3 MO [20].

The Arrb2 Morpholino 2 (Arrb2 MO2 5′-CGCACGGTTCCAAACGCACAGTAGG- 3′) and a Control Morpholino (Control MO) were purchased from Gene Tools LLC (OR, USA). The additional 5′UTR sequence of the *arrb2* mRNA has been submitted to Genebank (accession number KF831094).

The expression plasmids pCS2+ *pkcα-gfp*, pCS2+ *dn pkcα*, pCS2+ *camkII K42M*, pCS2+ *camkII T286D* were generously provided by Michael Kühl (University Ulm, Germany); pcDNA *HA-gβ1* (*HA-gnb1*), pcDNA *HA-gγ2* (*HA-gng2*) and pRK5 *β-ARKct* were provided by G. Schulte (Karolinska Institute, Stockholm, Sweden). The fragment encoding β-ARKct was subcloned into pCS2+. The open reading frames encoding Xenopus Dvl1, Dvl2 and Dvl3 were amplified by PCR and cloned into pCS2+ myc (R. Rupp, LMU Munich, Germany).

### Preparation of Cell Lysates, Immunoprecipitation, and Western Blotting

Cells were washed once with PBS and lysed in NP-40 buffer (20 mM Tris-HCl (pH 7.4), 150 mM NaCl, 2 mM EDTA, 1% NP-40) supplemented with complete Protease Inhibitor and PhosStop Phosphatase Inhibitor Cocktails (Roche, Mannheim, Germany) at 4°C. For embryo lysates, embryos were collected at the desired stage and lysed in the same lysis buffer. Animal Cap lysates were also prepared using the same lysis buffer. Lysates were cleared at 16,000× g for 10 min. For co-immunoprecipitation, lysates were incubated for 4 h at 4°C with the appropriate antibody and protein G-magnetic beads (Life Technologies, CA, USA). Immunoprecipitates were collected, washed four times with lysis buffer and eluted with SDS sample buffer. For Western blotting, proteins were visualized colorimetrically with NBT/BCIP.

### Antibodies

Commercial antibodies were obtained from Abcam, UK (mouse anti-Arrb2, rabbit anti-GFP; rabbit anti-HA, goat anti-myc), Cell Signaling Technology Inc., USA (rabbit anti-Arrb2, rabbit anti-myc, rabbit anti-Flag), ProteinTech Inc., USA (rabbit anti-Arrb2) and Santa Cruz Biotechnology Inc., USA (mouse anti-Gβ, mouse anti-β-Catenin, rabbit anti-Dvl2). The anti-tubulin β hybridoma developed by Michael Klymkowski and the anti-actin antibody developed by Jim Jung-Chin Lin were obtained from the Developmental Studies Hybridoma Bank developed under the auspices of the NICHD and maintained by The University of Iowa, Department of Biology, Iowa City, IA 52242. Secondary antibodies were anti-mouse-Alkaline Phosphatase and anti-rabbit-Alkaline Phosphatase (Cell Signaling Technology, Inc. USA), anti-mouse Cy3 (Jackson ImmunoResearch, PA, USA), anti-rabbit Alexa 488 and anti-mouse Alexa 647 (Life Technologies, CA, USA).

### Injection and Analysis of Xenopus laevis Embryos

RNA for microinjection was prepared using the mMessage mMachine Kit (Life Technologies, CA, USA). Injection amounts were 200 pg for *ca camkII* (*camkII T286D*), *dn camkII* (*camkII K42M*), and for *myc-arrb2*, *myc-arrb1*, *HA-gβ1* (*HA-gnb1*), and *HA-gγ2* (*HA-gng2*); injection amounts were 500 pg for *pkcα*, *dn pkcα*, *β-ARKct*, and 1 ng for *pkcα-gfp* and *fzd7*. For *ca rhoA* 5 pg and *ca rac1* 10 pg plasmid DNA were injected. Knock-down was achieved by injection of the following antisense Morpholino oligonucleotides: Dvl2 MO, Dvl1 MO, and Dvl3 MO (0.8 pmol each), Arrb2 MO1 (0.8 pmol), Arrb2 MO2 (0.8 pmol), Fzd7 MO (4 pmol). Pertussis Toxin (PTX) was added to the culture medium at 100 ng/ml.

Embryos were injected at the two-cell stage for Animal Cap explants or at the four-cell stage in both dorsal blastomeres for Keller explants and cultured until they reached stage 10 or 10.5, respectively.

Keller open face explants were prepared and cultured as described in [18]. Explants were scored as “fully elongated” if they showed >75% elongation, as “partially elongated” if elongation was between 25% and 75% and “not elongated” if explants showed less than 25% elongation when compared to fully elongated control explants. Immunofluorescence staining of Animal Cap explants was performed as described previously [21]. Photographs were taken on a Zeiss Apotome imaging system (Zeiss, Oberkochen, Germany).

## Results

### β-Arrestin2 is required for PKCα activation in Wnt/Ca^2+^ signaling

**Figure 1:**
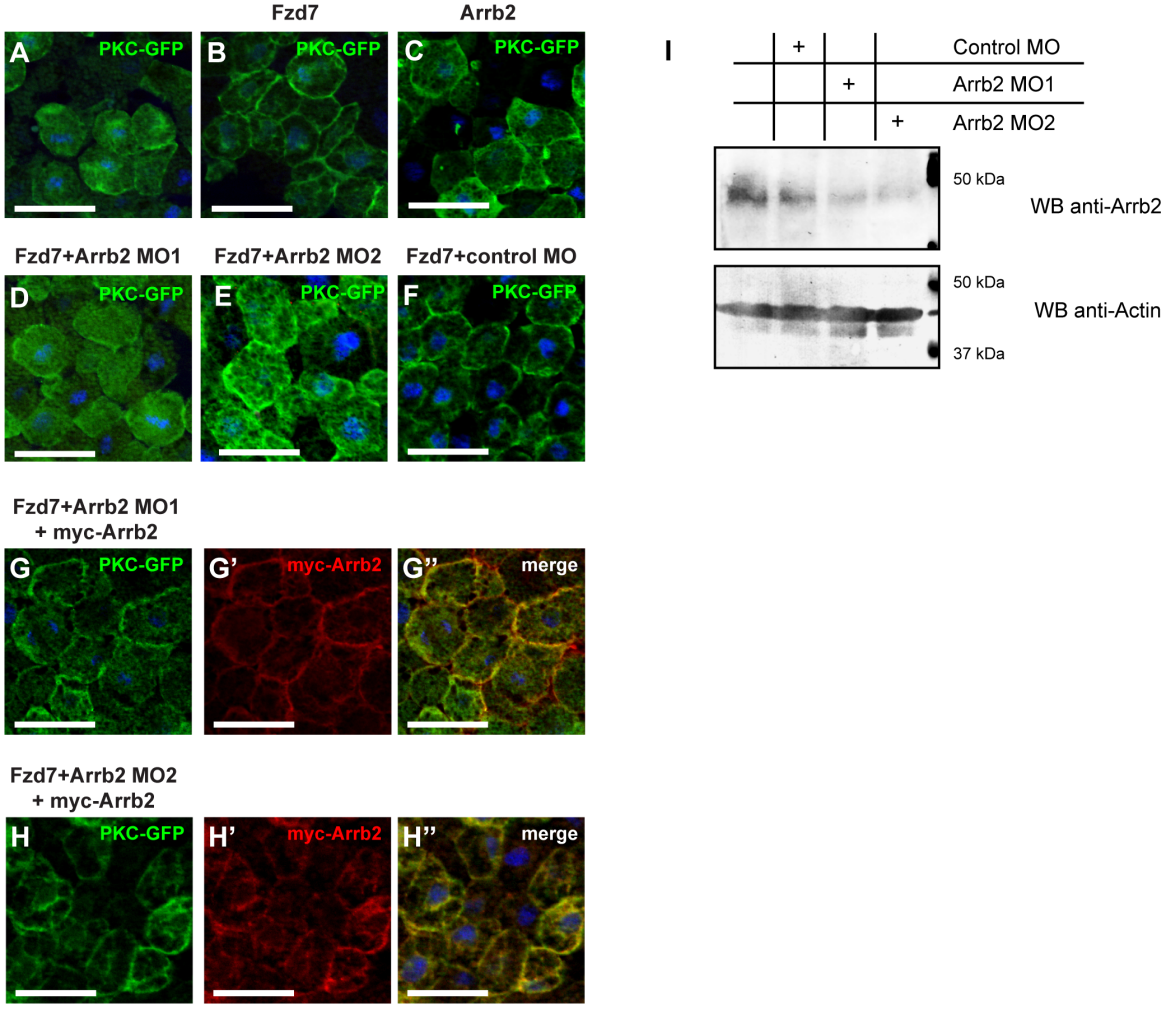
Arrb2 is required for membrane translocation of PKCα. Xenopus embryos were injected with 500 pg *pkcα-gfp* RNA and co-injected as indicated above the images. Animal Caps were prepared at stage 10 and immunostained as indicated. Nuclei were stained with Hoechst 33258 (blue). Images show representative results of at least three independent experiments with a minimum of six Animal Caps per experiment. Scale bars: 50 µm. (A) PKCα-GFP localized predominantly to the cytoplasm. (B) Co-injection of 1ng *fzd7* RNA induced PKCα-GFP translocation to the plasma membrane. (C) Overexpression of Arrb2 partially induced PKCα-GFP membrane translocation. Co-injection of *fzd7* mRNA with 0.8 pmol Arrb2 MO1 (D) or 0.8 pmol Arrb2 MO2 (E) blocked PKCα-GFP localization to the plasma membrane, while a Control MO had no effect (F). (G) The inhibitory effect of Arrb2 MO1 on PKCα-GFP membrane translocation was rescued by co-expression of myc-Arrb2 (anti-myc (red): G′, merge: G"). (H) Comparably, inhibition of PKCα-GFP membrane translocation by Arrb2 MO2 was restored by co-expression of myc-Arrb2 (anti-myc (red): H′, merge: H"). (I) Western Blot of Animal Cap lysates prepared from stage 10 embryos, which were either uninjected or injected with Control MO, Arrb2 MO1 or Arrb2 MO2 as indicated. Both Morpholinos efficiently downregulated proteins levels of endogenous Arrb2; an immunoblot for β-Actin is shown as loading control.

Wnt- or Frizzled-induced PKCα activation and translocation to the plasma membrane is an indicator for the activation of Wnt/Ca^2+^ signaling. We therefore investigated the role of β-Arrestin2 in Wnt/Ca^2+^ signaling by monitoring PKCα translocation in Xenopus Animal Cap explants. As expected from earlier studies [3], overexpression of *Xenopus* Frizzled 7 (Fzd7) induced a robust translocation of PKCα-GFP to the plasma membrane (Figure 1A, B). Interestingly, overexpression of β-Arrestin2 (Arrb2, Figure 1C) was also sufficient to induce partial membrane association of PKCα-GFP, likely by enhancing endogenous signaling. For further analysis knock-down of Arrb2 was achieved using two different translation-blocking antisense Morpholinos (Arrb2 MO1 [13] and Arrb2 MO2). Both Morpholinos repressed Frizzled 7 induced PKCα-GFP association with the plasma membrane to comparable extent (Figure 1D and Figure 1E). In contrast, a Control MO had no effect on Fzd7-mediated PKCα-GFP membrane localization (Figure 1F). The inhibition of Fzd7-induced PKCα-GFP translocation by Arrb2 MO1 or Arrb2 MO2 was rescued by co-injection of a Morpholino insensitive *myc-arrb2* RNA. Expression of myc-tagged Arrb2 restored PKCα-GFP localization to the plasma membrane in the presence of Arrb2 MO1 (Figure 1G, G′, G") or Arrb2 MO2 (Figure 1H, H′, H"), which confirmed the specificity of both Arrb2 Morpholino oligonucleotides used in this study. To determine the efficiency of protein depletion by the Morpholino oligonucleotides used herein, lysates of stage 10 Animal Cap explants were tested with an anti-Arrb2 antibody to detect the endogenous protein. Both Arrb2 MO1 and Arrb2 MO2 efficiently reduced endogenous Arrb2 protein levels in Animal Cap explants (Figure 1I), which confirmed the capability and efficiency of these Morpholino oligonucleotides to deplete endogenous Arrb2 in Animal Cap tissue.

The novel Arrb2 MO2 has been designed to bind in the 5′UTR of *arrb2* mRNA, thereby targeting a non-overlapping sequence compared to Arrb2 MO1 (Figure S1), which targets the translation start. Although *arrb2* and *arrb1* mRNA sequences are only 48% identical in the overall binding region of Arrb2 MO1, they share 12 out of 15 bases in the sequence surrounding the ATG codon (Figure S1). Therefore, the specificity of Arrb2 MO1 to Arrb2 as compared to Arrb1 was further confirmed using MO-sensitive GFP fusion constructs and by co-injecting PKCα-GFP expressing Animal Caps with Fzd7, Arrb2 Morpholinos and *arrb1* RNA. We observed that Arrb2 MO1 was not able to suppress translation of GFP constructs fused to the *arrb1* 5′ UTR, while it efficiently blocked translation when the *arrb2* 5′UTR was present (Figure S2A). In contrast to myc-Arrb2 (Figure 1G, H), myc-Arrb1 only partially restored Fzd7-induced PKCα-GFP translocation in Animal Caps injected with Arrb2 MO1 or Arrb2 MO2 (Figure S2B-S2E). These results further confirmed the specificity of the Arrb2 Morpholino oligonucleotides used here. Moreover, we found that mRNA levels of *arrb1* were on average more than 500 fold lower than those of *arrb2* in gastrula stage embryos (Figure S3).

Overall, we conclude that Arrb2 is required downstream of Frizzled 7 for membrane translocation and activation of PKC in the Wnt/Ca^2+^ pathway.

### β-Arrestin2 functionally interacts with the β subunit of trimeric G-proteins

**Figure 2:**
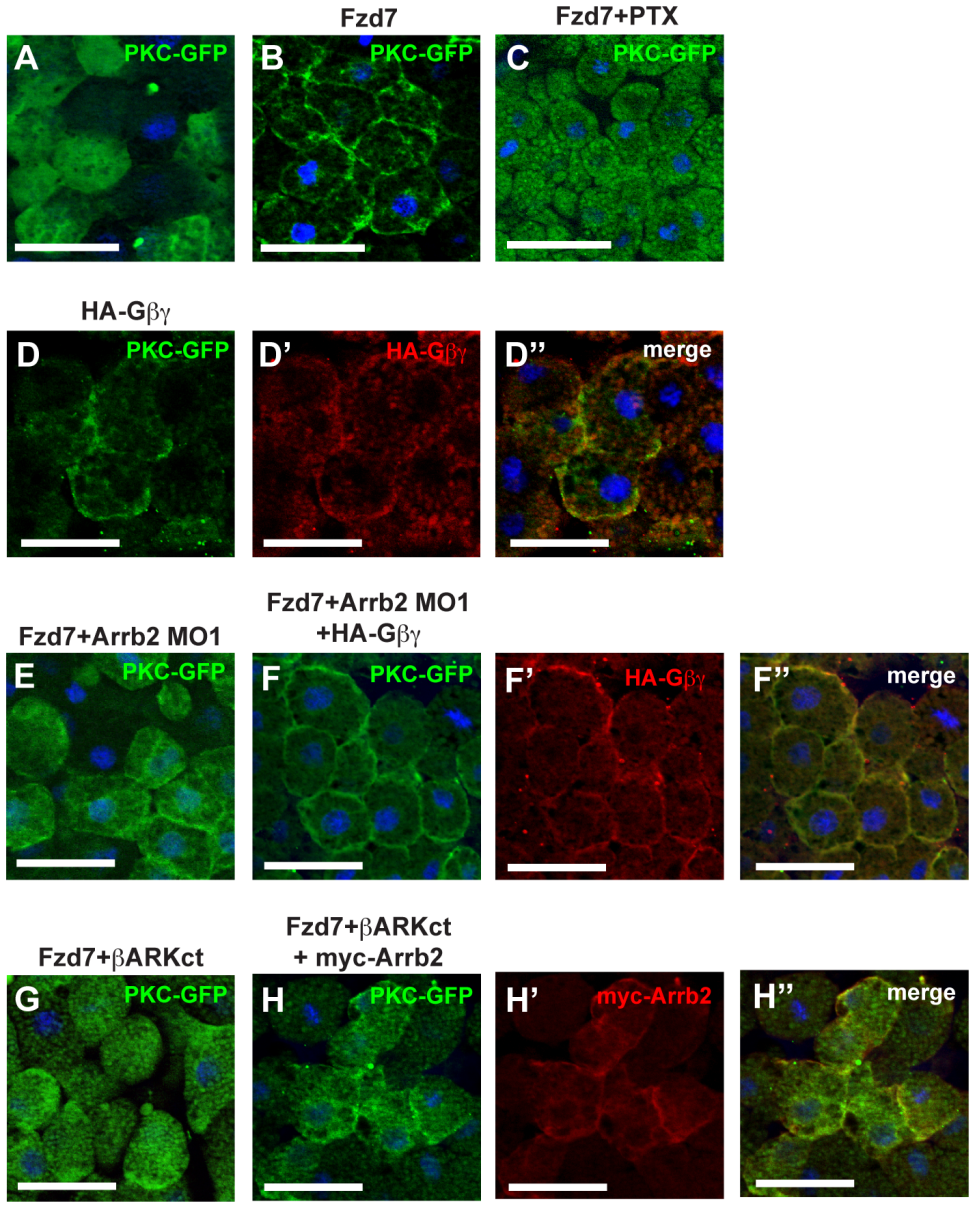
Arrb2 depends on Gβγ to induce membrane translocation of PKCα. Xenopus embryos were injected with 500 pg *pkcα-gfp* RNA and co-injected as indicated above the images. Animal Caps were prepared at stage 10 and immunostained as indicated. Nuclei were stained with Hoechst 33258 (blue). Images show representative results from at least two independent experiments with a minimum of six Animal Caps per experiment. Scale bars: 50 µm. (A) PKCα-GFP control, PKCα-GFP localized predominantly to the cytoplasm. (B) Co-injection of 1ng *fzd7* RNA induced PKCα-GFP translocation to the plasma membrane. (C) Treatment with PTX for 1 hour blocked Fzd7-induced PKCα-GFP translocation. (D) Overexpression of HA-Gβ and HA-Gγ subunits (indicated as HA-Gβγ) induced PKCα-GFP translocation (HA-Gβγ stained with anti-HA (red): D′, merge: D"). The inhibitory effect of Arrb2 MO1 (E) on PKCα-GFP membrane translocation was rescued by (F) co-injection of HA-Gβ and HA-Gγ mRNA (anti-HA (red): F′, merge: F"). (G) Overexpression of the Gβ-sequestering β-ARKct also blocked Fzd7 induced PKCα-GFP translocation. (H) Co-expression of myc-Arrb2 in Fzd7 and β-ARKct injected Animal Caps was not sufficient to rescue PKCα-GFP membrane translocation (anti-myc (red): H′, merge: H").

Frizzled-induced PKCα translocation requires activation of trimeric G-proteins and signal transduction mediated by the β- and γ-subunits (Gβ and Gγ) [16], which was recapitulated in our experiments by treatment of Animal Cap explants with Pertussis Toxin (PTX) for 1 hour (Figure 2A, B, C). Overexpression of HA-tagged Gβ1 and Gγ2 (HA-Gnb1 and HA-Gng2, respectively) resulted in PKCα activation and translocation in the HA-positive cells (Figure 2D, D′, D"). Interestingly, expression of the β and γ subunits of trimeric G-proteins was sufficient to revert the effect of β-Arrestin2 knock-down on PKC localization. Co-injection of Arrb2 MO1 blocked Fzd7-induced association of PKCα-GFP with the plasma membrane (Figure 2E and Figure 1C). PKCα translocation was restored by co-expression of HA-Gβ and HA-Gγ (Figure 2F, F′, F") indicating that Gβ/Gγ signaling activated PKCα independent or downstream of Arrb2. In the reverse experiment, endogenous Gβ activity was blocked by the Gβ-sequestering β-ARKct that is derived from the C-terminus of β-Adrenergic receptor kinase 2 (Adrbk2, [22]). β-ARKct efficiently inhibited Fzd7 induced PKCα-GFP translocation (Figure 2G). Co-expression of Arrb2 was not sufficient to rescue PKCα-GFP translocation in Animal Caps overexpressing β-ARKct (Figure 2H), although Arrb2 localization at the plasma membrane was not affected (Figure 2H′, H"). These results suggested that Arrb2 depends on Gβ signaling to induce PKC activation and translocation.

### β-Arrestin2 functionally interacts with Dishevelled in Wnt/Ca^2+^ signaling

In our previous studies, we have found that the interaction between β-Arrestin2 and Dvl is required for Wnt signal transduction in the Wnt/β-Catenin [13] and in the Wnt/PCP pathways [7]. In addition, it has been shown that overexpression of DvlΔDIX, a Dvl2 mutant that activates β-Catenin independent Wnt pathways, is capable of activating PKC and CamKII [15]. In Xenopus as in other vertebrates, three Dvl isoforms have been identified, and an initial study suggested that at least Xenopus Dvl1 and Dvl2 are functionally redundant while Dvl3 showed a different expression pattern and differential functionality in tadpole stage embryos [20].

**Figure 3:**
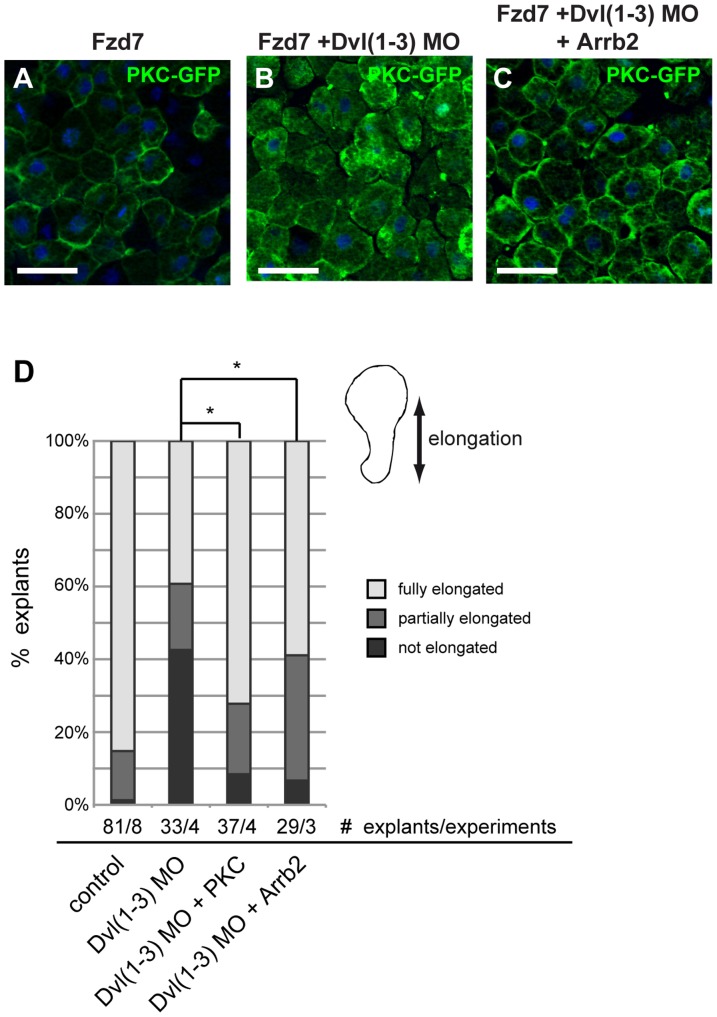
Arrb2 functionally interacts with Dvl in Wnt/Ca^2+^ signaling. Xenopus embryos were injected with 500 pg *pkcα-gfp* RNA and co-injected as indicated above the images. Animal Caps were prepared at stage 10 and immunostained as indicated. Nuclei were stained with Hoechst 33258 (blue). Images show representative results from at least two independent experiments with a minimum of six Animal Caps per experiment. Scale bars: 50 µm. Fzd7 induced PKCα-GFP translocation (A) was impaired by a triple knock-down of Dvl1, Dvl2 and Dvl3 (B). (C) Co-expression of Arrb2 partially rescued PKCα-GFP translocation in the triple Dvl knock-down. (D) Triple Dvl knock-down inhibited elongation of Keller open face explants. Co-injection of PCKα or Arrb2 mRNA rescued the CE phenotype of triple Dvl morphant explants. The average percentage of explants showing full (75-100%, light grey), partial (25-50%, medium grey) or no elongation (<25%, dark grey) from at least three independent experiments are shown. Asterisks indicate statistically significant deviations in the percentage of fully elongated explants (* p>0.95, t-test).

To analyze the functional interaction of Dvl and Arrb2 in Xenopus gastrulation, we first confirmed that Dishevelled was required for Fzd7-induced PKCα-GFP translocation to the plasma membrane in Animal Cap explants. To avoid redundancy and to obtain sufficient depletion of Dvl proteins, which are already present maternally [20], we simultaneously knocked-down Dvl1, Dvl2 and Dvl3. As expected, Fzd7-induced PKCα translocation was impaired in triple Dvl morphant embryos (Figure 3A, B). Interestingly, overexpression of Arrb2 rescued PKCα membrane translocation (Figure 3C), indicating that Arrb2 functionally interacted with Dvl in Wnt/Ca^2+^ signaling.

Convergent extension (CE) movements of the dorsal mesoderm are tightly regulated mass cell movements during vertebrate gastrulation, which require the activation of β-Catenin independent Wnt signaling pathways [23], [24]. Consistently, triple Dvl knock-down impaired elongation of Keller open face explants, which recapitulate CE movements of the dorsal mesoderm [25]. The majority of explants from triple Dvl morphant embryos showed either no elongation or only partial elongation (Figure 3D), and this elongation phenotype was rescued by co-injection of either *arrb2* or *pkcα* mRNA, confirming again the role of Dvl in Wnt/Ca^2+^ signaling and the functional interaction of Dvl and Arrb2.

To further characterize the role of β-Arrestin in Wnt/Ca^2+^ signaling and its function in the regulation of CE movements, we performed additional rescue experiments in Keller open face explants.

**Figure 4:**
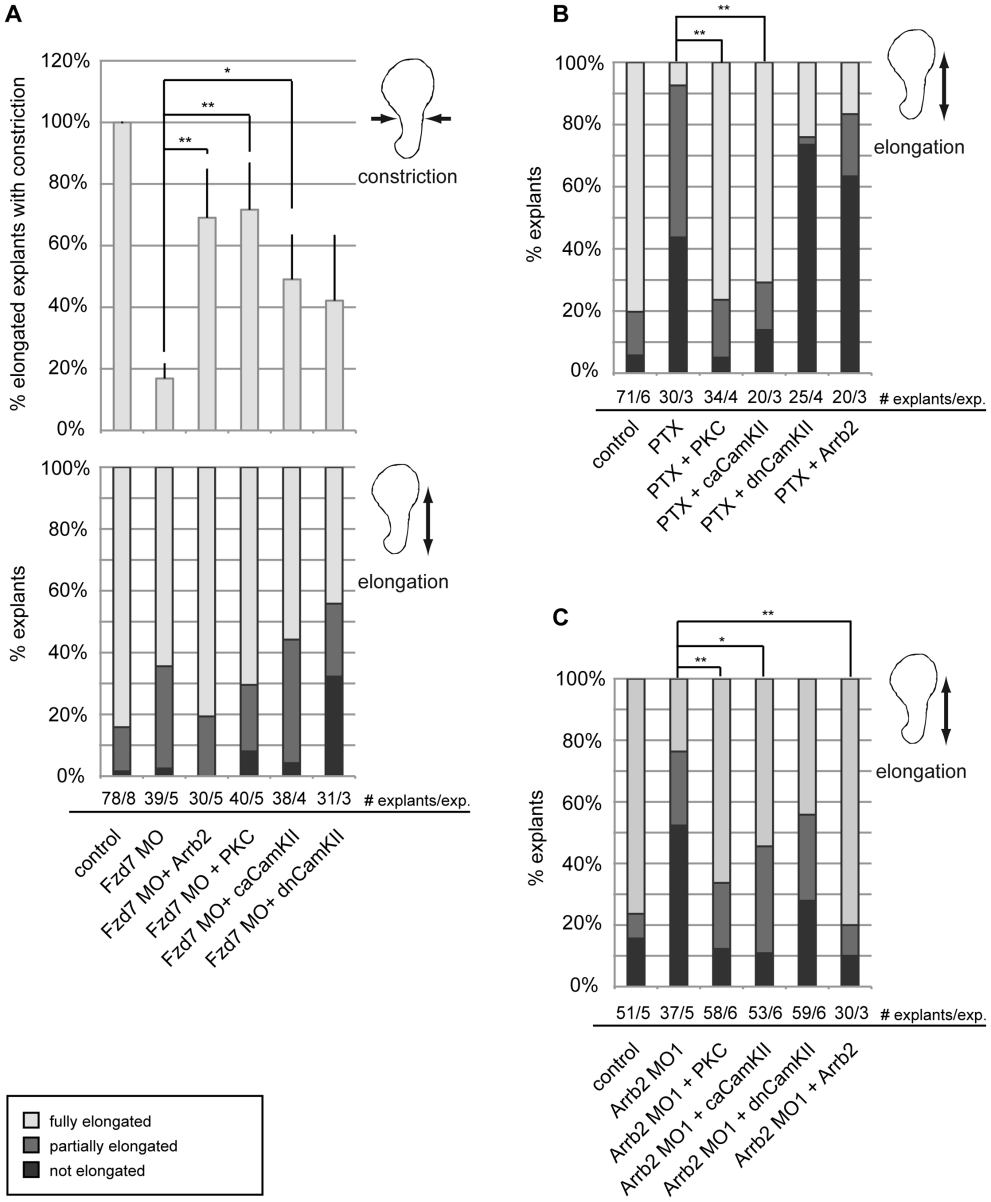
Arrb2 is required for CE movements downstream of Frizzled 7 and upstream of PKCα. Xenopus embryos were injected at 4-cell stage in the marginal zone of both dorsal blastomeres as indicated. CE movements in the dorsal mesoderm were monitored by elongation of Keller open face explants. The average percentage of explants showing full (75-100%, light grey), partial (25-50%, medium grey) or no elongation (<25%, dark grey) from at least three independent experiments (exp.) are shown. Asterisks indicate statistically significant deviations in the percentage of fully elongated explants (** p>0.99,* p>0.95, t-test). (A) Frizzled 7 knock-down had little effect on elongation (bottom graph) but impaired constriction. Only fully elongated explants (represented by light grey columns in bottom graph) were additionally scored for constriction. The percentage of elongated explants that showed normal constriction are shown in the upper graph as average values plus SEM. Co-injection of *arrb2* or *pkcα* mRNA fully rescued constriction and *ca camkII* mRNA partially rescued constriction. (B) PTX treatment impaired elongation of Keller open face explants. Co-injection of *pkcα* or *ca camkII* mRNA fully rescued explant elongation, while *dn camkII* or *arrb2* mRNA did not. (C) Knock-down of Arrb2 interfered with explant elongation and was rescued by co-injection of *pkcα*, *ca camkII* or a MO-insensitive *arrb2* RNA.

Explants from Frizzled 7 morphant embryos showed only mild elongation defects. However, in the vast majority of elongating explants, strong constriction defects were observed (Figure 4A). The Fzd7 morphant phenotype was fully rescued by co-injection of a mRNA encoding *arrb2* as well as by *pkcα* mRNA (Figure 4A), confirming the requirement of Arrb2 in Fzd7-mediated Ca^2+^-dependent signaling and the role of this branch of Wnt signaling in CE movements [26], [27]. In contrast to Fzd7 knock-down, treatment of Keller explants with PTX completely abrogated CE movements (Figure 4B). Scoring for constriction defects was not applicable in non-elongating explants, and we did not observe significant constriction defects under the conditions that rescue the PTX phenotype (not shown). PTX treatment was not effective in explants overexpressing PKCα or caCamKII, which further emphasized the role of Ca^2+^-dependent signaling in CE movements. However, overexpression of Arrb2 was not sufficient to restore CE movement in PTX-treated explants, which further supported the conclusion that β-Arrestin2 signaling to PKC was dependent on trimeric G-protein activity.

Previously, we have reported that β-Arrestin2 is required for convergent extension movements in Xenopus embryos as an effector in the Wnt/PCP pathway [7]. As shown in this previous study, knock-down of Arrb2 strongly inhibited explant elongation when compared to control explants (Figure 4C). Interestingly, Arrb2 loss-of-function (LOF) was again fully rescued by co-injection of *pkcα* mRNA or *ca camkII* mRNA and surprisingly, a partial, but not significant rescue was observed by co-injection of *dn camkII* mRNA. A Morpholino-insensitive *arrb2* mRNA also rescued the CE phenotype in Arrb2 morphant explants, which served as specificity control for the Morpholino-induced CE phenotype (Figure 4C).

Overall, these results demonstrated that β-Arrestin2 is required downstream of Fzd7 and upstream of PKC in Wnt/Ca^2+^ signaling. Moreover, we confirmed a functional interaction between the beta and gamma subunits of trimeric G-proteins and Dishevelled in signal transduction from Fzd7 to PKC in the regulation of CE movements during Xenopus gastrulation.

### Arrb2 forms a complex with Dvl and Gβγ

Frizzled receptors belong to the GPCR superfamily and have been shown to interact with Dishevelled [21], trimeric G-proteins [28], [29] and likely indirectly with β-Arrestin2 [30]. In addition, the β subunit of trimeric G-proteins has been found to interact with β-Arrestin1 [31] and Dvl [32]; and in our previous studies, we have found that the interaction between β-Arrestin2 and Dvl is required for Wnt signal transduction in the Wnt/β-Catenin [13] and in the Wnt/PCP pathways [7].

Moreover, our present results clearly demonstrated a functional interaction between β-Arrestin2, Gβγ and Dishevelled in early Xenopus embryos. These observations prompted us to further investigate the physical interaction among these proteins.

**Figure 5:**
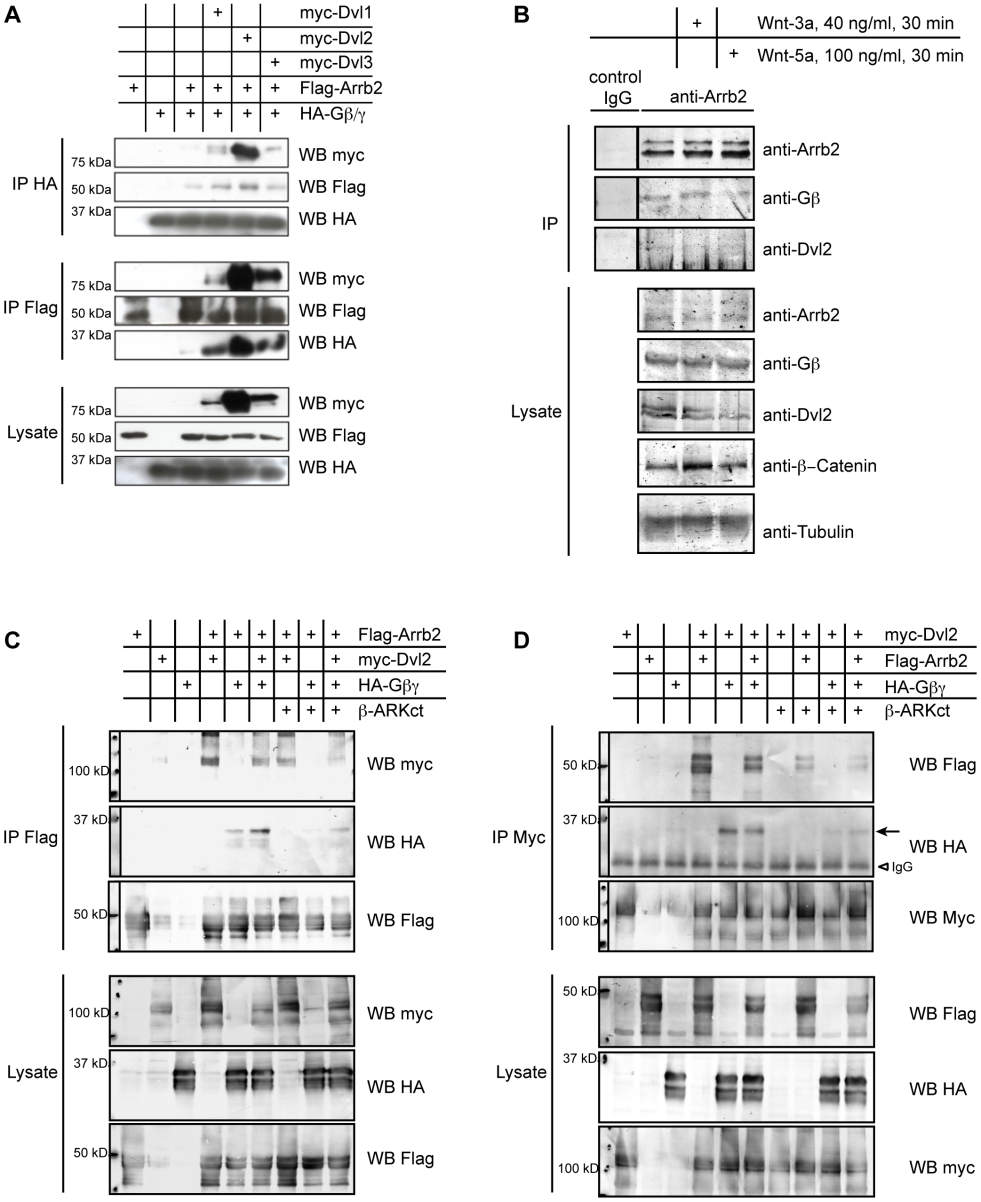
Arrb2 physically interacts with Gβ and Dvl. Epitope-tagged proteins were overexpressed, immunoprecipitated and detected by Western Blotting as indicated. (A) Flag-Arrb2 was co-expressed with a combination of HA-Gβ1 and HA-Gγ2 to allow the formation of Gβγ heterodimers (Gβγ). Co-expression of Dvl1, Dvl2 or Dvl3 enhanced the interaction between Arrb2 and Gβ1 in co-immunoprecipitation experiments from HEK 293T cells. (B) Endogenous Gβ and Dvl2 were detected in immunoprecipitates of endogenous Arrb2 from unstimulated and Wnt-stimulated HEK 293T cells. (C) Binding of Dvl2 to Arrb2 was also observed in the absence of exogenous Gβ. Myc-Dvl2 co-precipitated equally well with Flag-Arrb2 when Gβ1 and Gγ2 were overexpressed (Gβγ) as in the presence of the Gβ-sequestering β-ARKct in HEK 293T cells. By contrast, binding of Gβ1 to Arrb2 was impaired by β-ARKct and partially restored by co-expression of Dvl2. (D) When myc-Dvl2 was precipitated, the amount of Flag-Arrb2 and that of Gβ1 that co-precipitated with Dvl2 was significantly reduced by the co-expression of β-ARKct.

Co-immunoprecipitation experiments showed that all three Dvl isoforms formed a complex with Gβ1 and β-Arrestin2, and all enhanced binding of Gβ1 to β-Arrestin2, indicating that any Dvl is capable to form a trimeric complex with β-Arrestin2 and Gβ1 (Figure 5A). The endogenous existence of an Arrb2-Dvl-Gβ complex was confirmed by co-precipitation of Gβ and Dvl2 with Arrb2 from unstimulated and Wnt stimulated HEK293T cells (Figure 5B), although the overall low amounts of co-precipitated protein indicated that only a fraction of the respective proteins present in the cell are assembled in this complex.

Subsequently, we investigated if the interaction of Gβ1 with β-Arrestin2 or Dvl2 was affected by overexpression of the Gβ-binding C-terminal fragment of β-Adrenergic receptor kinase (β-ARKct). Overexpression of β-ARKct strongly interfered with the binding between Arrb2 and Gβ1 (Figure 5C). However, we still observed binding of Dvl2 to Arrb2 in the presence of β-ARKct, although reduced when compared to the binding in the absence of β-ARKct (Figure 5C). Co-expression of Dvl2 partially restored binding of Gβ1 to Arrb2, probably by enhancing the interaction between Arrb2 and residual free Gβ1 subunits (Figure 5C). We observed a similarly strong interference of β-ARKct with the interaction between Gβ and Dvl2 (Figure 5D), which was again partially restored by co-expression of Arrb2. In addition, the amount of Arrb2 that co-precipitated with Dvl2 was clearly reduced in the presence of β-ARKct, indicating that the interaction of Dvl2 and β-Arrestin2 did not strictly depend on Gβ signaling, but was significantly enhanced by Gβ.

## Discussion

### Arrb2 is required in Wnt/Ca^2+^ signaling

In addition to its previously described role as a mediator of signal transduction in the Wnt/β-Catenin and the Wnt/PCP pathway, we have demonstrated that β-Arrestin2 is also an essential effector in the Wnt/Ca^2+^ pathway that is required for the activation and membrane translocation of PKCα downstream of Frizzled 7. These results are in contrast to an earlier report [14], which stated that Arrb2 had no function in Wnt/Ca^2+^ signaling. Herein our results clearly show that Arrb2 knock-down achieved by two different Morpholinos targeting non-overlapping sequences in the *arrb2* mRNA blocked Fzd7-induced PKCα translocation to the plasma membrane and that overexpressed Arrb2 was capable to partially induce PKC activation. We also confirmed that the antisense Morpholinos used in this study efficiently reduced endogenous Arrb2 protein levels. Overexpression of Arrb2 rescued Arrb2 MO1 or Arrb2 MO2 and we have also observed a partial rescue by overexpression of Arrb1, which indicated partial functional redundancy. This result is consistent with a recent report showing partial redundancy of Arrb1 and Arrb2 in CE movements [33]. However, while Arrb2 is expressed at constantly high levels during gastrulation, Arrb1 mRNA levels decrease during this phase of development (Figure S3), suggesting that in gastrulating Xenopus embryos predominantly Arrb2 is required.

In addition to regulating PKCα membrane translocation, we have shown in this study that the convergent extension phenotype induced by Arrb2 knock-down was rescued by overexpression of PKCα or caCamKII and that Arrb2, PKCα or caCamKII rescued CE movements in Fzd7 morphant explants, further emphasizing the functional requirement of Arrb2 in Wnt/Ca^2+^ signaling.

### Functional and physical interaction of xArrb2 and Gβ with Dvl

In addition to Frizzled receptors, overexpression of Gβ and Gγ [16] induces PKCα translocation, and it has been shown that Dvl is also able to activate Wnt/Ca^2+^ signaling [15]. Here, we have investigated the functional interaction of these effector proteins in the Wnt/Ca^2+^ pathway.

Overexpression of Gβ and Gγ was sufficient to rescue PKCα activation in embryos injected with *fzd7* mRNA and Arrb2 MO1. In contrast, PKCα translocation blocked by inhibition of Gβ signaling was not rescued by overexpression of Arrb2, indicating that Gβ / Gγ signaling is either activated downstream of Frizzled and β-Arrestin or that β-Arrestin activity is Gβ-/ Gγ-dependent. Consistently, Arrb2 only weakly rescued CE movements in explants treated with PTX, but efficiently rescued the CE phenotype induced by Frizzled 7 knock-down. Therefore, we conclude that β-Arrestin2 interacts with trimeric G-proteins to activate PKCα, which is required for CE movements in Xenopus gastrulation.

In our own earlier studies, we have demonstrated that β-Arrestin physically interacts with Dvl [13]. Here we show that β-Arrestin2 was able to rescue PKCα membrane translocation and CE movements in triple Dvl morphant embryos, which is consistent with the previous studies and further confirmed the functional interaction of β-Arrestin2 and Dishevelled in Xenopus gastrulation.

Interaction between Arrb1 and Gβ as well as the interaction of Dvl2 with Gβ have also been reported [31], [32]. In light of the functional interactions of β-Arrestin 2, Dvl and Gβγ in Wnt/Ca^2+^ signaling, we hypothesized that these proteins formed a hetero-tetrameric complex. We have confirmed that Arrb2 binds to Gβ1. This result was expected because Gβ also binds to Arrb1 [31] and functional redundancy between Arrb1 and Arrb2 was indeed reported in a recent study by Kim and co-workers [33]. In addition to Arrb2, we observed that all Xenopus Dvl isoforms, Dvl1, Dvl2 and Dvl3 physically interacted with Gβ1. The interaction of each Dvl with Gβ1 was enhanced in the presence of Arrb2; moreover, the interaction between Arrb2 and Gβ1 was increased by any Dvl isoform, although Dvl2 seemed to have the strongest effect on Arrb2-Gβ1 binding.

Notably, Arrb2 rescued the Frizzled 7 knock-down and the triple Dvl phenotype in Xenopus gastrulation movements, but was not sufficient to restore CE movements in PTX-treated Keller explants or PKCα translocation to the plasma membrane in Animal Cap explants overexpressing the Gβ-sequestering C-terminus of β-ARK. Our results further showed that co-expression of Dvl strongly enhanced the interaction of β-Arrestin2 with Gβ while sequestering Gβ interfered with binding between β-Arrestin2 and Dvl2. Altogether, we conclude that activation of trimeric G-proteins downstream of Frizzled leads to the formation of a protein complex consisting of β-Arrestin2, the β and γ subunits of trimeric G-proteins and Dishevelled. In the Wnt/Ca^2+^ pathway, this complex likely triggers the activation of PKC and other Ca^2+^-dependent effector proteins.

Strikingly, β-Arrestin2 and Dishevelled also interact and are essential effectors in the Wnt/β-Catenin pathway and in Wnt/PCP signaling to the small GTPases RhoA and Rac1 [5], [7], [14], [34], [35]. Considering that Wnt/Ca^2+^ and Wnt/PCP signaling are often required simultaneously [8], [9] and are activated by the same Wnt ligand and Frizzled receptor [3]–[7] our findings support a close functional interaction of Wnt/Ca^2+^ and Wnt/PCP signaling in the regulation of CE movements in Xenopus embryos. Moreover, Frizzled receptors, which belong to the superfamily of G-protein coupled receptors, have recently been shown to interact with different types of trimeric G-proteins [29], and there is accumulating evidence that trimeric G-proteins play a role in apparently all Wnt/Frizzled signaling cascades [28], [29], [36]. We have shown here that the beta and gamma subunits of trimeric G-proteins, which dissociate from the alpha subunit upon activation, promote the interaction of β-Arrestin2 and Dishevelled, two proteins that have been identified as essential effectors in Wnt/β-Catenin [13], Wnt/PCP [7], [14] and in Wnt/Ca^2+^ signaling (this study). Therefore it can be hypothesized that complex formation involving β-Arrestin2 and Dishevelled and Gβγ might be a general mechanism of trimeric G-protein mediated activation of Wnt signaling cascades.

## Supporting Information

Figure S1 **Alignment of** ***arrb2*** **and** ***arrb1*** **sequences.** Alignment of the novel sequence stretch of the *arrb2* 5′UTR including the first 132 nucleotides of *arrb2* coding sequence to the corresponding sequences of *arrb2* mRNA (NCBI NM_001092112) and *arrb1* mRNA (NCBI NM_001094402) generated with ClustalW2. Asterisks indicate nucleotides conserved in all three sequences; morpholino binding regions are highlighted in light grey, the start codon is shown as bold and underlined.
(PDF)

Figure S2 *(A) Specificity of the Arrb2 MO1 antisense Morpholino oligonucleotide.* The 5′ UTR and coding sequence encoding amino acids 1–180 of *arrb2* and the corresponding sequences of the two *arrb1* pseudoalleles identified in gastrula stage embryos and termed *arrb1.a* and *arrb1.b* were were cloned in frame with GFP. All plasmids were co-injected with either Control MO or Arrb2 MO1 in 2-cell stage embryos and analyzed for expression of the GFP fusion proteins. An antibody against β-Tubulin served as loading control. *(B–E) Arrb1 only weakly influences PKCα-GFP membrane translocation.* Xenopus embryos were injected with 500 pg *pkcα-gfp* RNA and co-injected as indicated. PKC-GFP localization was analyzed in Animal Caps at stage 10 immunostained as indicated; nuclei stained with Hoechst 33258 (blue). Images show representative results from at least two independent experiments with a minimum of six Animal Caps per experiment. Scale bars: 50 µm. Overexpression of Arrb1 only weakly changed PKCα-GFP localization (Figure B, C). Consistently, co-injection of *myc-arrb1* RNA only partially restored PKCα-GFP membrane association in Animal Cap explants co-injected with Fzd7 and Arrb2 MO1 (D, D′: anti-myc, and D": merge). A comparable result was obtained when *myc-arrb1* RNA was co-injected with Fzd7 and Arrb2 MO2 (E, E′: anti-myc, E": merge).
(PDF)

Figure S3 **Detection of** ***arrb1*** **and** ***arrb2*** **transcripts in early Xenopus embryos.** Total RNA was extracted from Xenopus embryos of the indicated developmental stages, reverse transcribed and *arrb1*, *arrb2* and *ornithin decarboxylase* (*odc*) transcripts were amplified from the resulting cDNA. The upper panel shows the PCR fragments separated by agarose gelelectrophoresis from one representative experiment. The lower panel shows the corresponding real-time RT-PCR experiment using a different set of primer pairs (Illumina Eco Real Time PCR system; primer sequences are listed in Table S1). Transcription levels of *arrb1* and *arrb2* are plotted relative to *odc*.
(PDF)

Table S1 **RT-PCR Primer sequences.**
(PDF)
