# Supplementary material for: β-Arrestin Interacts with the Beta/Gamma Subunits of Trimeric G-Proteins and Dishevelled in the Wnt/Ca2+ Pathway in Xenopus Gastrulation
Source: PLoS One. 2014 Jan 29;9(1):e87132. doi: 10.1371/journal.pone.0087132 (PMC3906129; doi:10.1371/journal.pone.0087132)
Supplement: Figure S1 — Alignment of arrb2 and arrb1 sequences. Alignment of the novel sequence stretch of the arrb2 5′UTR including the first 132 nucleotides of arrb2 coding sequence to the corresponding sequences of arrb2 mRNA (NCBI NM_001092112) and arrb1 mRNA (NCBI NM_001094402) generated with ClustalW2. Asterisks indicate nucleotides conserved in all three sequences; morpholino binding regions are highlighted in light grey, the start codon is shown as bold and underlined. (PDF) [file pone.0087132.s001.pdf]

Figure S1

```
arrb2 5’UTR+CDS      ----------------------------------------GGCTTCCTACTGTGCGTTTGGAACCGTGCGCGACTCCGCGGAGGTGGGAT  50
NM_001092112 arrb2   ------------------------------------------------------------------------------------------
NM_001094402 arrb1   AGCAGCAGCAGCACATTAATCCCGTCCCCTCCTCTCCCTAATCTCCCCGGGATCTGCACAAAAGCACTCGCCTCTCTCGACCCCTGGCAC  90

arrb2 5’UTR+CDS      TGGAGCGCGTGAACCCAGAGCAAGCGGAGGAGCTG--------GGAAGATGGGGGAGAGGGCGGGGACCCGGGTTTTCAAGAAATCCAGC 132
NM_001092112 arrb2   -----------------------GAGGAGGAGCTG--------GGAAGATGGGGGAGAGGGCGGGGACCCGGGTTTTCAAGAAATCCAGC  59
NM_001094402 arrb1   TCCTGCACCTCCTGCCTGTCCTCCTGCACCTCCTGCCCCTCCTGCATGATGGGGGACAAAG---GAACCAGAGTATTTAAGAAGGCGAGT 177
                                              * *    ***        * * ********* *  *   * *** * ** ** *****  * **

arrb2  5’UTR+CDS     CCTAACTGCAAGCTCACCGTGTACCTTGGAAAGCGAGATTTTGTCGATCACCTGGATCGGGTTGATCCTGTGGATGGCGTCGTCCTTGTG 222
NM_001092112 arrb2   CCTAACTGCAAGCTCACCGTGTACCTTGGAAAGCGAGATTTTGTCGATCACCTGGATCGGGTTGATCCTGTGGATGGCGTCGTCCTTGTG 149
NM_001094402 arrb1   CCAAATGGAAAGCTGACTGTTTACTTGGGCAAGAGGGACTTTGTTGATCACGTAGACGTGGTGGATCCTGTGGATGGGGTGGTGTTGGTG 267
                     ** **  * ***** ** ** *** * ** *** * ** ***** ****** * **   *** ************** ** **  * ***
```
