# Supplementary figures and images for: β-Arrestin Interacts with the Beta/Gamma Subunits of Trimeric G-Proteins and Dishevelled in the Wnt/Ca2+ Pathway in Xenopus Gastrulation
Source: PLoS One. 2014 Jan 29;9(1):e87132. doi: 10.1371/journal.pone.0087132 (PMC3906129; doi:10.1371/journal.pone.0087132)

Figure S2

A

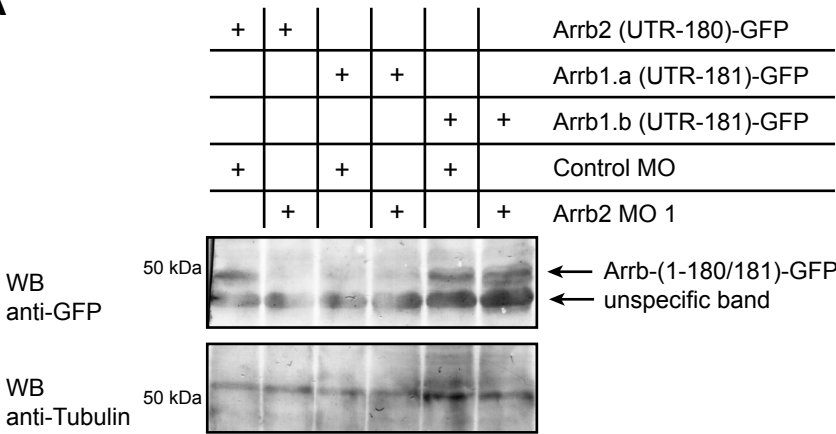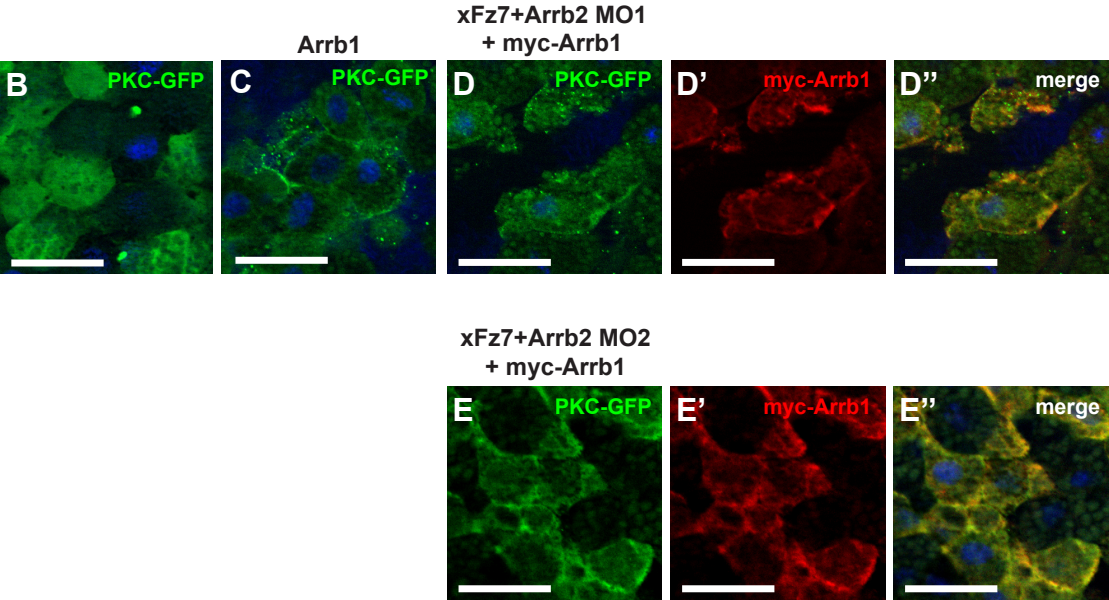

Supplement: Figure S2 — (A) Specificity of the Arrb2 MO1 antisense Morpholino oligonucleotide. The 5′ UTR and coding sequence encoding amino acids 1–180 of arrb2 and the corresponding sequences of the two arrb1 pseudoalleles identified in gastrula stage embryos and termed arrb1.a and arrb1.b were were cloned in frame with GFP. All plasmids were co-injected with either Control MO or Arrb2 MO1 in 2-cell stage embryos and analyzed for expression of the GFP fusion proteins. An antibody against β-Tubulin served as loading control. (B–E) Arrb1 only weakly influences PKCα-GFP membrane translocation. Xenopus embryos were injected with 500 pg pkcα-gfp RNA and co-injected as indicated. PKC-GFP localization was analyzed in Animal Caps at stage 10 immunostained as indicated; nuclei stained with Hoechst 33258 (blue). Images show representative results from at least two independent experiments with a minimum of six Animal Caps per experiment. Scale bars: 50 µm. Overexpression of Arrb1 only weakly changed PKCα-GFP localization (Figure B, C). Consistently, co-injection of myc-arrb1 RNA only partially restored PKCα-GFP membrane association in Animal Cap explants co-injected with Fzd7 and Arrb2 MO1 (D, D′: anti-myc, and D": merge). A comparable result was obtained when myc-arrb1 RNA was co-injected with Fzd7 and Arrb2 MO2 (E, E′: anti-myc, E": merge). (PDF) [file pone.0087132.s002.pdf]

Figure S3

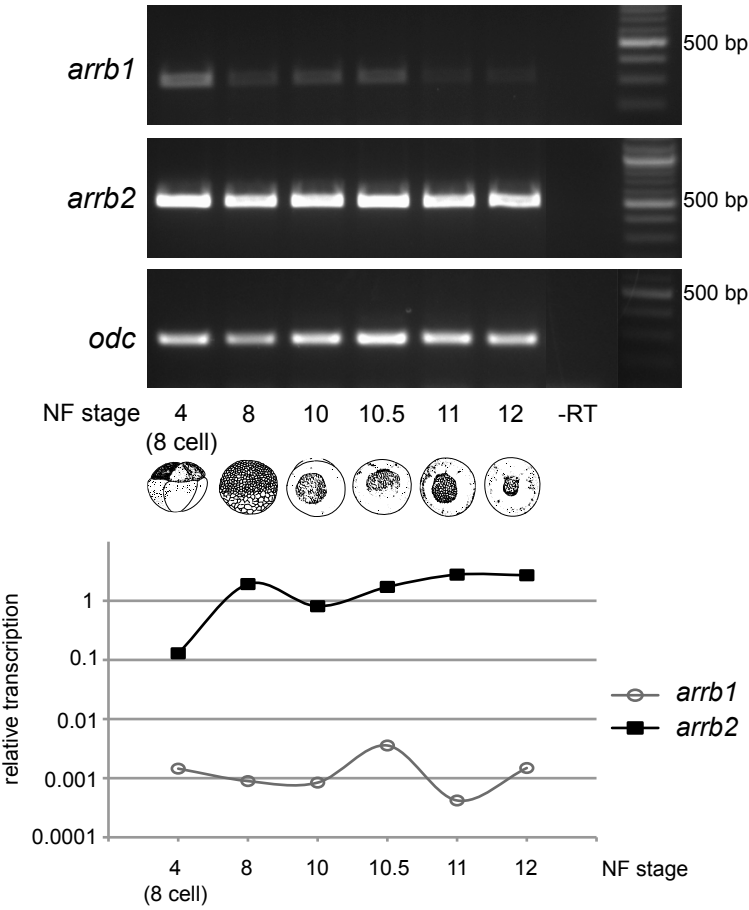

Supplement: Figure S3 — Detection of arrb1 and arrb2 transcripts in early Xenopus embryos. Total RNA was extracted from Xenopus embryos of the indicated developmental stages, reverse transcribed and arrb1, arrb2 and ornithin decarboxylase (odc) transcripts were amplified from the resulting cDNA. The upper panel shows the PCR fragments separated by agarose gelelectrophoresis from one representative experiment. The lower panel shows the corresponding real-time RT-PCR experiment using a different set of primer pairs (Illumina Eco Real Time PCR system; primer sequences are listed in Table S1). Transcription levels of arrb1 and arrb2 are plotted relative to odc. (PDF) [file pone.0087132.s003.pdf]
