# Supplementary material for: β-Arrestin Interacts with the Beta/Gamma Subunits of Trimeric G-Proteins and Dishevelled in the Wnt/Ca2+ Pathway in Xenopus Gastrulation
Source: PLoS One. 2014 Jan 29;9(1):e87132. doi: 10.1371/journal.pone.0087132 (PMC3906129; doi:10.1371/journal.pone.0087132)
Supplement: Table S1 — RT-PCR Primer sequences. (PDF) [file pone.0087132.s004.pdf]

Table S1

	forward Primer	reverse Primer
*arrb1*_1	tggaaagctgactgtttacttgg	atctcaaaggtgaatggataggc
*arrb2*_1	ctgacaggtctctgcacctagaa	caaaggtcgtgagatgttctctg
*odc*_1	gatgggctggatcgtatcgt	tggcagcagtacagacagca
*arrb1*_2	aggaattcagtgcgtttggtaat	caaatatcagcatactggcgaac
*arrb2*_2	ctatgaaattcgtgccttctgtg	attgcctctccgtggtaatagag
*odc*_2	cattgcagagcctgggagata	tccactttgctcattcaccataac
